# Black soybean seed coat polyphenols prevent AAPH-induced oxidative DNA-damage in HepG2 cells

**DOI:** 10.3164/jcbn.16-48

**Published:** 2016-12-06

**Authors:** Yasukiyo Yoshioka, Xiu Li, Tianshun Zhang, Takakazu Mitani, Michiko Yasuda, Fumio Nanba, Toshiya Toda, Yoko Yamashita, Hitoshi Ashida

**Affiliations:** 1 Organization of Advanced Science and Technology, Kobe University, Kobe, Hyogo 657-8501, Japan; 2 Graduate School of Agricultural Science, Kobe University, Kobe, Hyogo 657-8501, Japan; 3 Research & Development Department, Fujicco Co., Ltd., Kobe, Hyogo 650-8558, Japan

**Keywords:** procyanidin, 8-OHdG, ROS, LC-MS/MS, hepatocytes

## Abstract

Black soybean seed coat extract (BE), which contains abundant polyphenols such as procyanidins, cyanidin 3-glucoside, (+)-catechin, and (−)­epicatechin, has been reported on health beneficial functions such as antioxidant activity, anti-inflammatory, anti-obesity, and anti-diabetic activities. In this study, we investigated that prevention of BE and its polyphenols on 2,2'-azobis(2-methylpropionamide) dihydrochloride (AAPH)-induced oxidative DNA damage, and found that these polyphenols inhibited AAPH-induced formation of 8-hydroxy-2'-deoxyguanosine (8-OHdG) as a biomarker for oxidative DNA damage in HepG2 cells. Under the same conditions, these polyphenols also inhibited AAPH-induced accumulation of reactive oxygen species (ROS) in the cells. Inhibition of ROS accumulation was observed in both cytosol and nucleus. It was confirmed that these polyphenols inhibited formation of AAPH radical using oxygen radical absorbance capacity assay under the cell-free conditions. These results indicate that polyphenols in BE inhibit free radical-induced oxidative DNA damages by their potent antioxidant activity. Thus, BE is an effective food material for prevention of oxidative stress and oxidative DNA damages.

## Introduction

Reactive oxygen species (ROS) are generated during cell metabolism, and they play important roles in cell signaling and homeostasis. ROS is well-established physiological molecules and its production controlled by an antioxidative defense system. Excess production of ROS and/or defective cellular antioxidant systems has been implicated in many pathological conditions including chronic liver injury and fibrogenesis.^(1–3)^ Cells are naturally provided with an extensive array of protective enzymes to scavenge ROS,^(4)^ but the ROS level is dramatically increased by occurring environmental stresses under the certain conditions.^(5)^ Oxidants also produce enormous oxidative DNA damage, which is involved in the formation of tumors, and the onset of diabetes and other degenerative diseases.^(6,7)^ 8-Hydroxy-2'-deoxyguanosine (8-OHdG) is one of the biomarkers for oxidative DNA damage and carcinogenesis.^(8,9)^ Formation of 8-OHdG has been shown to lead to G: C-to-T: A transversion mutations that are prevalent in mutated in tumor suppressor genes.^(10,11)^ Recently, the 8-OHdG was detected and quantified with high sensitivity by a liquid chromatography- tandem mass spectrometry (LC-MS/MS).^(12)^ Quantitative determination of 8-OHdG has been performed in cultured cells, animal organs and human samples.^(9)^

Black soybean, a soybean cultivar with a black pigment in its seed coat, has been used widely as a food for hundreds of years in Asia. Its seed coat also has been used as a folk medicine. Black soybeans are used as a functional food for prevention of various diseases and counteracting toxicity.^(13)^ Many researchers have focused on the bioactivity of black soybean because the seed coat abundantly contained various polyphenols such as catechins, anthocyanins, procyanidins and other flavonoids.^(14)^ Polyphenols in black soybean seed coat have been reported to possess various health beneficial functions, such as antioxidant activity,^(15)^ anti-inflammatory responses, anti-obesity and anti-diabetic activity.^(16,17)^ Our previous study has demonstrated that black soybean seed coat polyphenols inhibited benzo[a]pyrene-induced micro nucleus assay in mice liver and cultured hepatocytes.^(14)^ Recent research shows black soybean possesses a strong inhibitory effect against low density lipoprotein oxidation *in vitro*.^(18)^ Black soybean also shows stronger 2-diphenyl-1-picrylhydrazyl radical scavenging activity, ferric reducing antioxidant power, and hydrophilic-oxygen radical absorbance capacity (H-ORAC) than yellow soybean.^(18,19)^ In the present study, we investigated the protective effects of polyphenols containing in BE against 2,2'-azobis(2-methylpropionamide) dihydrochloride (AAPH)-induced 8-OHdG formation and ROS accumulation in HepG2 cells.

## Materials & Methods

### Chemicals

**Fig. 1:**
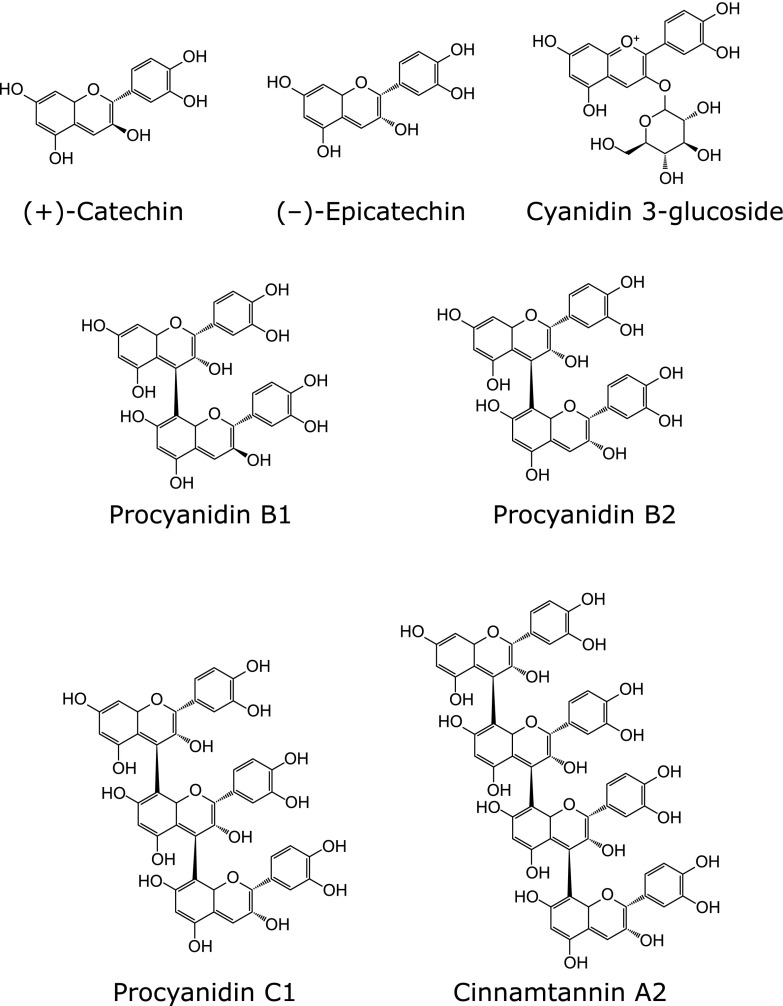
Chemical structures of C3G and procyanidins contained in BE.

AAPH and 8-OHdG were purchased from Wako Pure Chem. Ind. (Osaka, Japan). 2',7'-Dichlorodihydrofluorescin diacetate (DCFH_2_-DA) and 8-mercaptoguanosine (8-SHG) were purchased from Sigma Aldrich (St. Louis, MO). BE, procyanidins-rich BE, cyanidin 3-glucoside (C3G), procyanidin B1, procyanidin B2, procyanidin C1, and cinnamtannin A2 were obtained from Fujicco Co., Ltd. (Kobe, Japan). (+)-catechin and (–)-epicatechin were purchased from Nagara science (Gifu, Japan). The black soybean seed coat extract (BE) used in this study contained 6.2% epicatechin, 39.7% procyanidin (6.1% dimers, 3.4% trimers, and 0.5% tetramers), and 9.2% cyanidin 3-glucoside.^(16)^ The conposition of procyanidin rich-BE were 13.8% epicatechin, 66.8% procyanidin (11.8% dimers, 7.8% trimers, and 3.1% tetramers), and 0.9% cyanidin 3-glucoside. The chemical structures of the major polyphenols in BE are shown in Fig. 1. All other reagents were of the highest grade commercially available.

### Cell culture and treatments

HepG2, human hepatoma cell line, was obtained from American Type Culture Collection (Manassas, VA) and cultured in Dulbecco’s modified Eagle’s medium (DMEM; Nissui Pharmaceutical, Tokyo, Japan) containing 10% (v/v) fetal bovine serum (FBS; Sigma), 2 mM l-glutamine, 100 U/ml penicillin and 100 µg/ml streptomycin under a humidified atmosphere of 5% CO_2_ at 37°C. Cultures were allowed to reach 80–90% confluence before starting experiments. The cells were seeded on culture dishes or microplates, and incubated with either test sample or vehicle for 24 h. After the cells were washed with phosphate-buffered saline (PBS) twice to remove BE or compounds, oxidative stress was then induced by adding 25 mM AAPH to the cells for 1 h and 3 h to measure ROS accumulation and 8-OHdG formation, respectively.

### Preparation of DNA sample

The treated HepG2 cells were submitted to isolation of the nuclei according to the previous method with some modifications.^(20)^ Briefly, the cells were scraped with 100 µl of PBS, washed three times with ice-cold PBS, and centrifuged at 1,000 × *g* for 10 min at 4°C. The pellet was gently homogenized in an ice cold 5 mM 4-(2-hydroxyethyl)-1-piperazineethanesulfonic acid (HEPES) buffer (pH 7.5) containing 0.25 M sucrose and 0.5 mM ethylene glycol tetraacetic acid, and again centrifuged at 1,000 × *g* for 10 min at 4°C. The cells were washed with the buffer for another twice under the same conditions. Then, the nuclei were lysed with 400 µl of tris(hydroxymethyl)aminomethane-ethylenediaminetetraacetic acid (Tris-EDTA, TE) buffer (10 mM Tris-HCl, pH 7.4, and 1 mM EDTA) containing 0.5% sodium dodecyl sulfate, and treated with a final concentration of 0.5 mg/ml of ribonuclease A (Sigma Aldrich) for 30 min at 50°C. After adding 0.5 M NaCl, DNA was precipitated in 50% isopropanol and centrifuged at 17,000 × *g* for 15 min. The pellet was dissolved in 35 mM sodium acetate containing 1 mM EDTA, mixed with a final concentration of 500 nM 8-SHG as an internal standard, and treated with 0.2 mg/ml of nuclease P1 (Sigma Aldrich) at 37°C for 30 min. After the reaction was stopped by adding 0.1 M Tris-HCl (pH 7.5), DNA solution (90 µl) was treated with 3 units (10 µl of 30 units) of alkaline phosphatase (Sigma Aldrich) for 1 h at 37°C, and centrifuged at 17,000 × *g* for 10 min at 4°C. The supernatant was filtered through a 0.45 µm membrane filter, and DNA concentration in the supernatant was quantified using the Nano Drop^®^ ND-1000 spectrophotometer (NanoDrop, Wilninton, DE).

### Measurement of 8-OHdG with LC-MS/MS and HPLC

 8-OHdG was detected and quantified by LC-MS/MS (LCMS-8040, Shimadzu, Kyoto, Japan) using electrospray ionization (ESI). A L-column 2 ODS 1.5 ID × 150 mm (Chemicals Evaluation and Research Institute, Tokyo, Japan) column was employed to separate the compounds in the LC system. The mobile phases were 0.1% formic acid in distilled water (v/v, solvent A) and acetonitrile (solvent B). The flow rate was set at 0.2 ml/min, and the column temperature was held at 40°C. The gradient elution program was set as follows: 0–1 min, 0% solvent B; 1–13 min, 0–80% solvent B; 13–14 min, 80–100% solvent B. The LC column was then equilibrated for 5 min. Positive ESI in multiple reactions monitoring mode was used to record the signals of the target analytes. The ion source and other MS/MS parameters were optimized by injecting approximately 10 ng/µl of standard solution by an injector program or by using Automation Optimizer Software supplied by Shimadzu Corporation with the mobile phase of 0.1% formic acid in distilled water and acetonitrile (v/v, 50:50) at a flow rate of 0.2 ml/min. To achieve the maximum sensitivity, the mass spectrometer parameters for the standards were also optimized. Detection of 8-OHdG and 8-SHG was carried out under the positive ESI mode. Peak area of 8-OHdG was normalized by that of the 8-SHG. The amounts of dG were measured using HPLC with the following analytical conditions: column, L-column 2 ODS 1.5 ID × 150 mm maintained at 40°C; mobile phase, a mixed solvent of 6.5% methanol and 93.5% 20 mM potassium phosphate buffer (pH 4.5) containing 0.1 mM EDTA; flow rate, 1.0 ml/min. The dG was detected using a diode array detector (SPD-M20A, Shimadzu). The amount of dG was almost constant levels from 0.41 to 0.45 nmol/µg DNA in HepG2 cells. To evaluate the DNA oxidation, the ratio of 8-OHdG and dG was calculated.^(20)^

### Measurement of intracellular ROS accumulation in HepG2 cells

Intracellular ROS accumulation in HepG2 cells was monitored using fluorescent probe DCFH_2_-DA. Briefly, HepG2 cells were seeded on a 96-well plate and pretreated with or without test compounds for 24 h. Oxidative stress was induced by incubating 25 mM AAPH into the culture medium for 20, 40, and 60 min. At the end of the incubation, the cells were washed twice with PBS, and 5 µM DCFH_2_-DA was added to the cells and incubated for another 30 min. After the cells were washed twice with PBS again, they were treated with 1 µg/ml 4',6-diamidino-2-phenylindole (DAPI) for nuclear counter-staining. The amounts of DCF reflecting ROS accumulation and that of DAPI were quantified using a fluorescence microplate reader (Perkin Elmer, Aaltham, MA) at 485/535 nm and 355/460 nm, respectively.

### Fluorescence microscopy

Intracellular distribution of ROS was detected in the DCFH_2_-DA-treated cells under the fluorescence microscopy analysis. Briefly, HepG2 cells were seeded in a 24-well plate and pretreated with or without procyanidins-rich BE and BE for 24 h. Oxidative stress was induced by incubating 25 mM AAPH into the culture medium for 1 h. At the end of incubation, the cells were washed twice with PBS, and 5 µM DCFH_2_-DA was added to the cells and incubated for another 30 min. After the cells were washed twice with PBS again, they were immobilized with 4% paraformaldehyde solution for 20 min, and treated with 1 µg/ml DAPI for counter staining of nucleus. Fluorescence of DCF and DAPI was measured at 485/535 nm and 355/460 nm, respectively, using the FSX100 microscopy (Olympus, Tokyo, Japan).

### Measurement of AAPH radical scavenging capacity

The oxygen radical scavenging capacity was measured using the H-ORAC assay with some modifications.^(21)^ H-ORAC assay is based on the fluorescence decay of fluorescein as a reference substance, after adding AAPH as a peroxy radical generator. Briefly, BE, procyanidins-rich BE, C3G, (+)-catechin, (–)-epicatechin, procyanidin B1, procyanidin B2, procyanidin C1, and cinnamtannin A2 were dissolved in dimethyl sulfoxide and added to a 96-well microplate followed by the addition of 70 nM fluorescein in 75 mM phosphate buffer (pH 7.4) and incubated for 15 min at 37°C. As the positive and negative controls, Trolox in dimethyl sulfoxide and dimethyl sulfoxide alone were used, respectively. After adding of 25 mM AAPH in 75 mM phosphate buffer (pH 7.4), fluorescence was recorded every 2 min for 120 min with excitation and emission wavelength at 485 nm and 535 nm, respectively, by the microplate reader (Perkin Elmer). Results were expressed as Trolox equivalent.

### Statistical analysis

The data are expressed as the mean ± SE from at least three independent determinations for each experiment. Dunnett’s test was used to determine significant differences between the treated and control groups. The level of statistical significance was set to *p*<0.05.

## Results

### Determination of quantitative method of 8-OHdG by LC-MS-MS

To quantify 8-OHdG in AAPH-treated HepG2 cells, we established a measurement method using LC-MS/MS. The multiple reaction monitoring channels for 8-OHdG and 8-SHG (internal standard) were found to be 283.90/168.05 and 317.10/185.05 m/z, respectively, corresponding to the [M+H]^+^ precursor ion and product fragment. The detection limit (signal-to-noise ratio>10:1) was determined to be 1 nM 8-OHdG (data not shown). In order to confirm the 8-SHG can be used as the internal standard to measure 8-OHdG from the cell samples, 8-OHdG and 8-SHG were simultaneously measured. It was found that the retention time of them was 4.91 min and 5.06 min, respectively, and there is no 8-SHG existing in DNA sample from the cells (data not shown). The calibration curve of 8-OHdG showed a high linearity from 1 nM to 1 µM. These results indicated that 8-SHG can be used as the internal standard to detect the formation of 8-OHdG in DNA samples from HepG2 cells. Using the LC-MS/MS method, we examined the protective effect of BE and its polyphenols on the AAPH-induced 8-OHdG formation in HepG2 cells.

### BE polyphenols inhibited AAPH-induced 8-OHdG formation in HepG2 cells

**Fig. 2:**
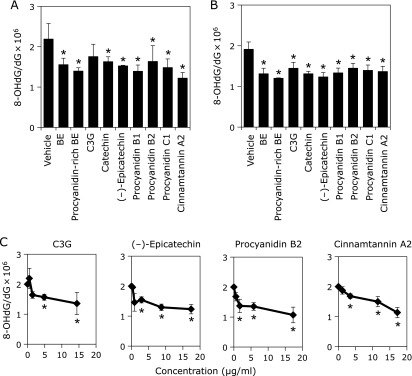
Polyphenols in BE inhibited AAPH-induced 8-OHdG formation in HepG2 cells. (A) BE and procyanidins-rich BE at 2.9 µg/ml (equivalent of 10 µM (–)-epicatechin) were treated to HepG2 cells for 24 h. C3G, (+)-catechin, (–)-epicatechin, procyanidin B1, procyanidin B2, procyanidin C1, and cinnamtannin A2 at 10 µM were treated to HepG2 cells for 24 h. (B) The extracts and its polyphenols at 2.9 µg/ml were treated to HepG2 cells for 24 h. (C) C3G, (–)-epicatechin, procyanidin B2, and cinnamtannin A2 at several concentrations were treated to HepG2 cells for 24 h. Then, these cells were exposed to 25 mM AAPH for 3 h. After nuclei were isolated from these cells, DNA was extracted, purified and subjected to LC-MS/MS for and HPLC analysis for 8-OHdG and dG, respectively. The value of 8-OHdG was evaluated as 8-OHdG/dG × 10^6^ in nuclei. Three independent experiments were performed. *Significant difference from the 8-OHdG level in DNA sample from the corresponding vehicle-treated cells by Dunnett’s test (*p*<0.05).

The intranuclear 8-OHdG level was significantly elevated in the presence of AAPH compared to that in the absence of AAPH (data not shown). AAPH-induced 8-OHdG formation was also measured after treatment with BE and procyanidins-rich BE at 2.9 µg/ml (equivalent of 10 µM (–)-epicatechin), and C3G, (+)-catechin, (–)-epicatechin, procyanidin B1, procyanidin B2, procyanidin C1, and cinnamtannin A2 at 10 µM to to HepG2 cells for 24 h. BE, procyanidins-rich BE, (+)-catechin, (–)-epicatechin, procyanidin B1, procyanidin B2, procyanidin C1, and cinnamtannin A2 significantly decreased the AAPH-induced 8-OHdG formation in HepG2 cells (Fig. 2A). However, C3G did not show significant inhibition. All polyphenols at 2.9 µg/ml significantly inhibited the formation of 8-OHdG with the almost equal potency (Fig. 2B). Selected 4 compounds, C3G, (–)-epicatechin, procyanidin B2, and cinnamtannin A2 suppressed the formation of 8-OHdG in a dose-dependent manner (Fig. 2C).

### BE polyphenols inhibited AAPH-induced ROS accumulation in HepG2 cells

**Fig. 3:**
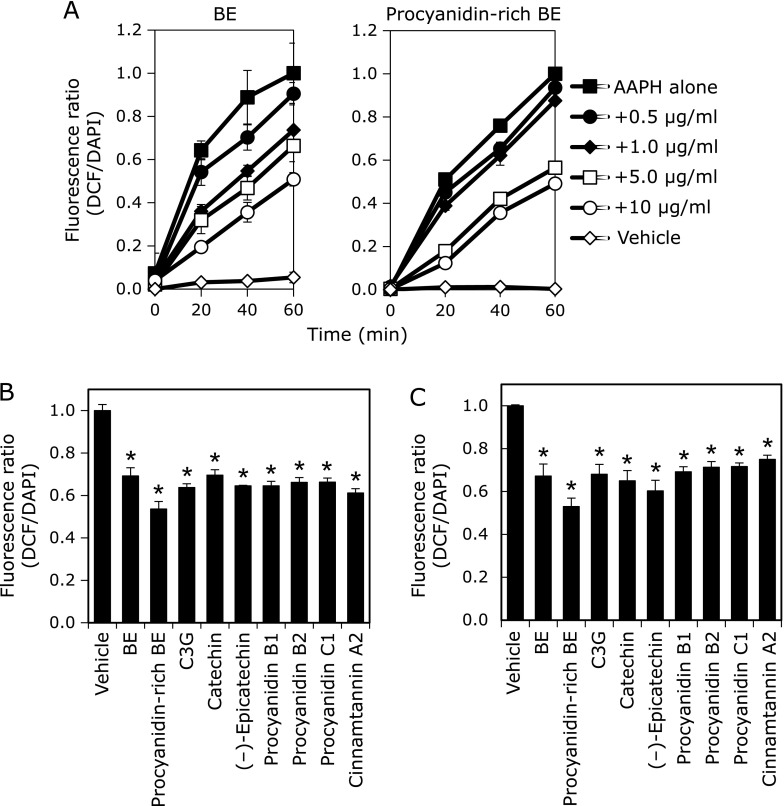
BE polyphenols inhibited AAPH-induced ROS accumulation in HepG2 cells. (A) BE and procyanidins-rich BE at 0.5, 1.0, 5.0 and 10 µg/ml were treated to HepG2 cells for 24 h. The cells were exposed to 25 mM AAPH and incubated for 20, 40 and 60 min. (B) BE and procyanidins-rich BE at 2.9 µg/ml (equivalent of 10 µM (–)-epicatechin) were treated to HepG2 cells for 24 h. C3G, (+)-catechin, (–)-epicatechin, procyanidin B1, procyanidin B2, procyanidin C1, and cinnamtannin A2 at 10 µM were treated to HepG2 cells for 24 h. (C) Polyphenols containing in BE at 2.9 µg/ml were treated to HepG2 cells for 24 h. Then, these cells were incubated with 5 µM DCFH_2_-DA for 30 min and stained nuclear with DAPI. Fluorescence DCF and DAPI were measured at 485/535 nm and 355/460 nm, respectively, with a microplate reader. Three independent experiments were performed. *Significant difference from the ROS level in cell lysate from the corresponding control cells by Dunnett’s test (*p*<0.05).

To determine the inhibitory effect of BE and its polyphenol against the AAPH-induced intracellular ROS accumulation, the level of ROS was measured using DCFH_2_-DA probe. AAPH induced DCFH oxidation in a time-dependent manner (Fig. 3A). It was found that BE and procyanidins-rich BE significantly inhibited AAPH-induced ROS accumulation in a time- and dose-dependent manner. We also examined the inhibitory effect of polyphenols contained in BE on AAPH-induced ROS accumulation in HepG2 cells, and found that all compounds tested significantly decreased ROS accumulation at 10 µM (Fig. 3B) and at 2.9 µg/ml (Fig. 3C).

### BE and procyanidins-rich BE inhibited AAPH-induced nucleus ROS accumulation in HepG2 cells with fluorescent microscopy

**Fig. 4:**
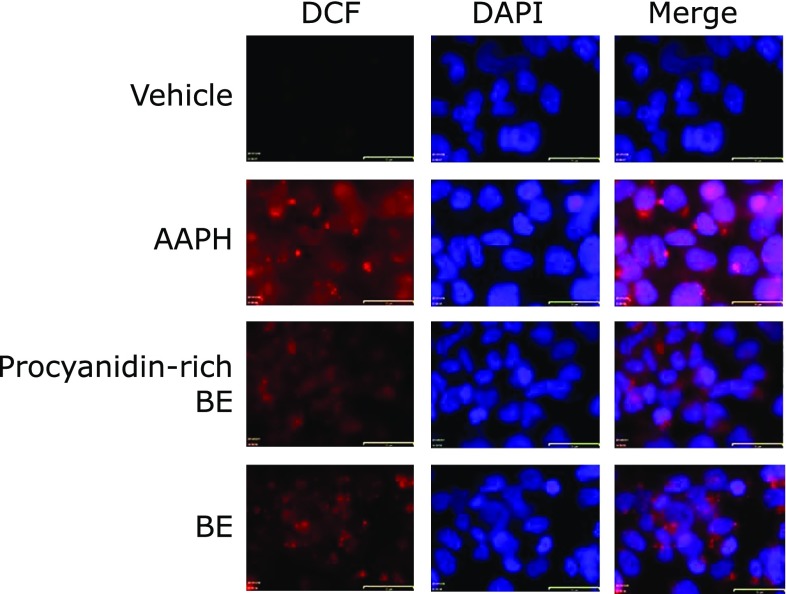
AAPH-induced intracellular and intranuclear ROS accumulation was inhibited by BE and procyanidins-rich BE in HepG2 cells. BE and procyanidins-rich BE at 5 µg/ml were treated to HepG2 cells for 24 h. The cells were incubated with 25 mM AAPH for 1 h and then 5 µM DCFH_2_-DA for 30 min. After loading with DCFH_2_-DA, the cells immobilized and stained nuclear with DAPI. Fluorescence DCF (red) and DAPI (blue) was visualized at 485/535 nm and 355/460 nm, respectively, with a microscopy. The scale bars are 32 µm.

Cellular localization of ROS accumulation and inhibitory effect of BE and procyanidins-rich BE was evaluated by a fluorescent microscopy. AAPH-induced ROS accumulation was observed in both cytosol and nuclear, and it was substantially decreased after treatment with BE and procyanidins-rich BE (Fig. 4).

### BE polyphenols are potent radical scavengers

**Fig. 5:**
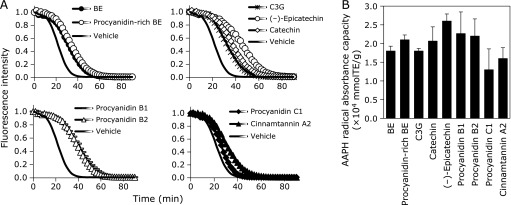
AAPH radical scavenging capacity of BE polyphenols. (A) Fluorescence decay curve and (B) ORAC values of BE, procyanidins-rich BE and their polyphenols in the presence of fluorescein and AAPH. Mixture of test samples and 70 nM fluorescein were incubated for 15 min at 37°C. After the addition of 25 mM AAPH, fluorescence was monitored every 2 min on the micro plate reader. The ORAC value was calculated from the net area under the fluorescence decay curve using several concentrations of trolox as a standard.

To confirm antioxidant activity of BE and its polyphenols, H-ORAC assay was introduced, and AAPH was used as the peroxy radical source. ORAC values of BE, procyanidins-rich BE, C3G, (+)-catechin, (–)-epicatechin, procyanidin B1, procyanidin B2, procyanidin C1, and cinnamtannin A2 were 1.8 × 10^4^, 2.1 × 10^4^, 1.8 × 10^4^, 2.1 × 10^4^, 2.6 × 10^4^, 2.3 × 10^4^, 2.2 × 10^4^, 1.3 × 10^4^, and 1.6 × 10^4^ µmol TE/g, respectively (Fig. 5). ORAC value of procyanidins-rich BE, in which C3G content is less than 1%, was higher than that of BE. (–)-Epicatechin showed the highest ORAC value among used polyphenols. ORAC values of dimer procyanidins, procyanidin B1 and procyanidin B2, were higher than BE, while those of trimer procyanidin C1 and tetramer cinnamtannin A2 were lower than BE.

## Discussion

In this study, we have demonstrated that procyanidins containing in BE prevented 8-OHdG formation and ROS generation in HepG2 cells, indicating that procyanidins are strong radical scavengers. Using a quantitative analysis of 8-OHdG using LC-MS/MS, (+)-catechin, (–)-epicatechin, and procyanidins prevented AAPH-induced 8-OHdG formation in HepG2 cells (Fig. 2). Furthermore, they inhibited AAPH-induced ROS production in the cells (Fig. 3). Inhibition of ROS production was observed in both nucleus and cytosol in BE and procyanidins-rich BE treated cells by fluorescent microscopy analysis (Fig. 4). Moreover, polyphenols in BE showed a potent AAPH radical scavenging activity estimated by H-ORAC assay (Fig. 5). Our findings demonstrated that procyanidins, the major polyphenols in BE, are potent radical scavengers. Thus, these radical scavengers will be contributed to prevent from the 8-OHdG formation in hepatocytes.

ROS-induced oxidative DNA damage has been implicated in mutagenesis and carcinogenesis. This damage is mainly accompanied by the formation of 8-OHdG.^(6)^ Formation of 8-OHdG has been reported in several carcinoma cells.^(22–24)^ Moreover, 8-OHdG in human muscle, lung and intestine are increasing with aging, smoking habit and chronic diseases.^(25–27)^ Since 8-OHdG is one of the biomarkers for carcinogenesis, establishment of a high-sensitive analytical method for 8-OHdG is important. Our method for detection of 8-OHdG using LC-MS/MS was found to be sensitive and precise. Owing to the complex nature of the sample extract, it is not uncommon for each method to require the several labor-intensive clean-up steps prior to analysis. For these reasons, isotopic labeled internal standards are necessary for quantification by mass spectroscopy. However, isotopic labeled internal standards are not stable and would not be cost-effective for a clinical laboratory.^(28)^ Recently, 8-SHG as a cheap and readily available non-isotopic internal standard was established for LC-MS/MS assay to detect 8-OHdG in urine.^(29)^ In the present study, we confirmed that 8-SHG also can be used as an internal standard for detection of 8-OHdG in the cells system. Using the developed method, we found that (+)-catechin, (–)-epicatechin, and procyanidins inhibited the 8-OHdG formation. Procyanidins in beverages, vegetables and fruits have been reported to prevent oxidative stress and DNA damage. For example, procyanidins in wine protect against H_2_O_2_-induced oxidative stress in Fao cells,^(30)^ procyanidins from apple juice reduce oxidative DNA damage *in vivo*,^(31)^ and grape seed procyanidins-rich extract protects DNA from H_2_O_2_-induced oxidative stress.^(32)^ Therefore, procyanidins will be the main active compounds in BE that suppressed DNA damage.

It is known that various ROS such as hydroxyl radical, superoxide anion, and hydrogen peroxide, caused oxidative stress. The antioxidant defense system diminished ROS through mainly antioxidant enzymes such as catalase, superoxide dismutase, glutathione peroxidase, glutathione- *S*-transferase (GST), and glutathione reductase.^(33,34)^ We previously reported that BE and its component procyanidins and C3G enhanced protein expression of GST family in HepG2 cells.^(35)^ Thus, prevention effect of BE and its polyphenols against ROS accumulation were two ways: a direct radical scavenging activity and inducing GSTs expression indirectly. ROS accumulation due to mitochondrial disorder causes destabilization of the mitochondrial and/or nuclear genome.^(35,36)^ BE and its polyphenols effectively suppressed AAPH-induced ROS accumulation in not only cytoplasm but also nuclear (Fig. 4). This suggests that the direct radical scavenging activity is mainly contributed to the inhibition of oxidative DNA damage in nucleus of HepG2 cells by polyphenols in BE.

H-ORAC method is based on the inhibition of the peroxy-radical-induced oxidation initiated by thermal decomposition of AAPH and is well used to determine the antioxidant capacity in food extracts and their compounds. For instance, seed coats of black soybeans varieties were analyzed to antioxidant activity such as ferric reducing antioxidant power, DPPH radical scavenging activity, and H-ORAC.^(37)^ A study showed that Chardonnay grape seed powder displayed the strongest antioxidant capacity (1,076.4 µmol TE/g) estimated by H-ORAC method among seed powders from raspberry, blueberry, cranberry and grapes.^(38)^ In this study, H-ORAC value of BE was 1.8 × 10^4^ µmol TE/g indicating that radical scavenging activity of BE is much higher than that of other seed coat extracts.

Main polyphenols in BE extract are procyanidins. Compared with treatment of each 10 µM procyanidin, cinnamtannin A2, a tetramer procyanidin, strongly inhibited 8-OHdG formation and ROS accumulation. On the other hands, compared with treatment of each 2.9 µg/ml procyanidin, all procyanidins and their monomers showed the inhibitory effect of same strength. Therefore, we suggested that inhibition of procyanidins against 8-OHdG formation and ROS accumulation depended on numbers of hydroxyl moiety, but not numbers of flavan-3-ol structure. When BE was orally given to the mice at 1 g/kg body weight, cinnamtannin A2 was not detected in the plasma. Aglycone forms of epicatechin, procyanidin B2, and procyanidin C1 were detected 1.94 ± 0.61, 3.22 ± 1.46, and 0.15 ± 0.19 µM, respectively (Yamashita Y, *et al.*, submitted). Previous studies also showed epicatechin, catechin, procyanidin B1, procyanidin B2, and procyanidin C1, but not cinnamtannin A2, were detected in plasma after intragastric injection of cacao or apple procyanidins.^(39,40)^ From these results, cinnamtannin A2 may not contribute inhibitory effect of oxidative DNA damage *in vivo*. Monomers and dimers of flavan-3-ols may contribute the inhibitory effect at least in part. Further study is needed to clarify the inhibitory effect of procyanidins on oxidative DNA damage *in vivo*.

In conclusion, polyphenols containing in BE prevented AAPH-induced 8-OHdG formation by possessing strong radical scavenging activity in HepG2 cells. Thus, BE is an effective food material for prevention of oxidative DNA damages.

## Abbreviations

AAPH: 2,2'-azobis (2-methylpropionamide) dihydrochloride
BE: black soybean seed coat extract
C3G: cyanidin 3-glucoside
EDTA: ethylenediaminetetraacetic acid
HEPES: 4-(2-hydroxyethyl)-1-piperazineethanesulfonic acid
H-ORAC: hydrophilic-oxygen radical absorbance capacity
LC-MS/MS: liquid chromatography-tandem mass spectrometry
8-OHdG: 8-hydroxy-2'-deoxyguanosine
PBS: phosphate-buffered saline
ROS: reactive oxygen species
Tris: tris(hydroxymethyl)aminomethane
